# Radical Scavenging Capacity and In Vitro Cytoprotective Effects of Great Salt Lake-Derived Processed Mineral Water

**DOI:** 10.3390/antiox13101266

**Published:** 2024-10-18

**Authors:** Takayuki Mokudai, Seiko Nakagawa, Hiroyasu Kanetaka, Kazuo Oda, Hiroya Abe, Yoshimi Niwano

**Affiliations:** 1 Institute for Materials Research, Tohoku University, 2-1-1 Katahira, Aoba-ku, Sendai 980-8577, Japan; tmokudai@tohoku.ac.jp; 2 Joining and Welding Research Institute, Osaka University, 11-1, Mihogaoka, Ibaraki 567-0047, Japan; abe.hiroya.jwri@osaka-u.ac.jp; 3 Measurement and Analytical Technology Group, Tokyo Metropolitan Industrial Technology Research Institute, 2-4-10, Aomi, Koto-ku, Tokyo 135-0064, Japan; nakagawa.seiko@iri-tokyo.jp; 4 Graduate School of Dentistry, Tohoku University, 4-1, Seiryo-machi, Aoba-ku, Sendai 980-8575, Japan; hiroyasu.kanetaka.e6@tohoku.ac.jp; 5 Greenheart International Co., Ltd., 2-36-29, Tarumicho, Suita 564-0062, Japan; k.oda@greenheart-intl.com; 6 Faculty of Nursing, Shumei University, 1-1 Daigaku-cho, Yachiyo 276-0003, Japan

**Keywords:** Great Salt Lake, mineral water, radical scavenging, cytoprotection

## Abstract

The Great Salt Lake, located in Utah, USA, is a saltwater lake with no outlet and is surrounded by vast mountains and salt deserts. We aimed to use Great Salt Lake-derived processed mineral water (hereafter termed as GSL-MW) for maintaining oral health. Therefore, we examined its radical scavenging activity as an antioxidant and its cytoprotective effect on human gingival fibroblasts (hGFs). The scavenging activity against O_2_^•−^ radicals was determined by an electron spin resonance (ESR)-spin trapping technique using two kinds of O_2_^•−^ generation systems; however, we could not reach any concrete conclusion because of the interference caused by GSL-MW in both systems. Detection of ·OH radicals using the ESR-spin trapping technique and kinetic analyses using double-reciprocal plots (corresponding to Lineweaver–Burk plots that are used to represent enzyme kinetics) revealed that GSL-MW has the ability to scavenge ·OH radicals. GSL-MW also showed a weak 2,2-diphenyl-1-picrylhydrazyl (DPPH; a stable radical)-scavenging activity. Regarding the cytoprotective effects, subconfluent hGFs pretreated with 10× and 100× dilutions of GSL-MW for 3 min and then exposed to harsh environmental conditions, such as pure water or 100 μM H_2_O_2_ for 3 min, showed enhanced cell viability rate. Moreover, 10× and 100× dilutions of GSL-MW reduced oxidative damage in confluent hGFs exposed to 12.5 and 25 mM H_2_O_2_. Our findings show that GSL-MW has antioxidant potential and cytoprotective effects on hGFs, suggesting that GSL-MW can be used to maintain oral health.

## 1. Introduction

An increasing amount of evidence suggests that spa mineral water has in vivo antioxidative properties, such as modulation by antioxidant enzymes [1,2,3], improvement of body redox status [4], and reduction of plasma or serum reactive oxygen species (ROS) levels [3,5]. In addition, a recent in vitro study showed that some mineral waters enhanced the antioxidant capacity (associated with ROS, reactive nitrogen species, and glutathione levels and superoxide dismutase activity) at the cellular level and cell proliferation rate [6]. The antioxidant capacity of spa mineral water is, at least in part, associated with its health benefits.

The Great Salt Lake, located in Utah, USA, is a saltwater lake with no outlet and is surrounded by vast mountains and salt deserts. It is a mysterious lake with salinity levels nine times higher than that of seawater. The Great Mineral Series (GMS), produced by Greenheart International Co., Ltd. (Suita, Japan), concentrates the mineral components present in water from the Great Salt Lake by evaporating the water under sunlight over a period of one year, after which the crystallized salt is removed (99.5%) to produce a mineral concentrate. In this study, we focused on the antioxidant capacity of food-grade GMS (hereafter termed Great Salt Lake-derived processed mineral water, GSL-MW) from the perspective of free radical scavenging. Spa mineral waters have been reported to empower wound healing strategies by enhancing the proliferation of fibroblasts via their antioxidant properties [6]; therefore, we examined the protective effects of GSL-MW on human gingival fibroblasts (hGFs) exposed to stress conditions. In previous studies, we searched for agents that could be used for maintaining oral health and found a potent antioxidant with the ability to protect hGFs that were pretreated with the antioxidant for as short as 1–3 min and subsequently exposed to harsh environmental conditions [7,8]. Thus, in the present study, we pretreated hGFs with GSL-MW for 3 min to examine the cytoprotective effects of GSL-MW on hGFs.

## 2. Materials and Methods

### 2.1. Test Material and Reagents

GSL-MW was obtained from Greenheart International Co. Ltd. (Suita, Japan). The mineral composition of GSL-MW and the osmolarity of GSL-MW dilutions (determined by the freezing point depression method) are shown in Table 1 and Table 2, respectively. Reagents were purchased from the following sources: 2,2-diphenyl-1-picrylhydrazyl (DPPH) from Tokyo Chemical Industry (Tokyo, Japan); 5,5-dimethyl-1-pyrroline N-oxide (DMPO) and xanthine oxidase (XOD) from Labotec (Tokyo, Japan); hypoxanthine (HPX), superoxide dismutase (SOD; from bovine erythrocytes), allopurinol, and 4-hydroxy-2,2,6,6-tetramethylpiperidine N-oxyl (TEMPOL) from Sigma-Aldrich (St. Louis, MO, USA); 3-(4,5-dimethyl-2-thiazolyl)-2,5-diphenyl-2H-tetrazolium bromide (MTT) and ethylenediamine-N,N,N′,N′-tetraacetic acid trisodium salt trihydrate (EDTA-3Na) from Dojindo Laboratories (Kumamoto, Japan); hydrogen peroxide (H_2_O_2_), riboflavin, and dimethyl sulfoxide (DMSO) from Wako Pure Chemical Industries (Osaka, Japan). All other reagents were of analytical grade.

### 2.2. Electron Spin Resonance (ESR)-Spin Trapping Technique for Determination of Superoxide Anion Radicals (O_2_^•−^) Generated by the HPX–XOD Cell-Free System

The assay used in this study was identical to that described previously [9,10]. In brief, a mixture of 50 μL of 2 mM HPX, 30 μL of DMSO, 50 μL of aqueous sample, 20 μL of 4.5 M DMPO, and 50 μL of 0.4 U/mL XOD was transferred to an ESR spectrometry cell, and the DMPO–OOH spin adduct (derived from O_2_^•−^) was quantified 97 s after the addition of XOD using an X-band ESR spectrometer (JES-FA-100; JEOL, Tokyo, Japan). The concentration of the DMPO–OOH spin adduct was determined using the method described in a previous report [11]. Briefly, the areas of DMPO–OOH spectra were compared with that of a 20 μM TEMPOL spectrum used as a standard, measured under identical settings, using Digital Data Processing (JEOL, Tokyo, Japan). The measurement conditions for ESR were as follows: field sweep, 330.5–340.5 mT; field modulation frequency, 100 kHz; field modulation width, 0.07 mT; amplitude, 200 or 250; sweep time, 2 min; time constant, 0.1 s; microwave frequency, 9.42 GHz; microwave power, 4 mW. In the experiment involving kinetic analyses using double reciprocal plots, different concentrations of DMPO and GSL-MW, the reference agent SOD, a superoxide scavenger, or allopurinol, an XOD inhibitor, were added to the reaction system, as described previously [12].

### 2.3. ESR-Spin Trapping Technique for Determination of Superoxide Anion Radicals (O_2_^•−^) Generated by the Photoexcitation of Riboflavin

A reaction mixture consisting of 4.5 mL of aqueous sample, 250 μL of 4.5 M DMPO, 5 mL of 400 mM EDTA·3Na, and 250 μL of 100 μM riboflavin was transferred to an ESR spectrometry cell, and the cell was placed in an X-band ESR spectrometer (JES-FA-200; JEOL) equipped with a sample mixer (ES-SM2; JEOL). The cell placed in the ESR spectrometer was irradiated with light emitted from a xenon lamp (ES-UXL500 25A; Ushio Inc., Tokyo, Japan) for 10 s through an L-39 filter (transmitting light of wavelengths above 390 nm). Immediately after irradiation, the DMPO–OOH spin adduct was analyzed using the ESR spectrometer. The measurement conditions for ESR were as follows: field sweep, 335.3 ± 5 mT; field modulation width, 0.05 mT; time constant, 0.1 s; microwave power, 10 mW; manganese (Mn) intensity, 800; measurement time, 1 min. ESR spectrometry was conducted at the Tokyo Metropolitan Industrial Technology Research Institute (other ESR spectrometric analyses were conducted at Tohoku University).

### 2.4. ESR-Spin Trapping Technique for Determination of Hydroxyl Radicals (·OH) Generated by Fenton Reaction

To determine the hydroxyl radical (·OH) scavenging activity of GSL-MW using the Fenton reaction, ferrous sulfate (FeSO_4_) was used as a source of ferrous ions; FeSO_4_ was added to hydrogen peroxide (H_2_O_2_) to induce the Fenton reaction, as shown in the following equation:

H_2_O_2_ + Fe^2+^ → ·OH + OH^−^ + Fe^3+^

An aliquot (80 μL) of different dilutions of GSL-MW or different concentrations of DMSO (a ·OH scavenger used as a reference agent) was mixed with 50 μL of 2 mM H_2_O_2_, 20 μL of 8.9 M DMPO, and 50 μL of 0.2 mM FeSO_4_ for 10 s. Immediately after mixing, the mixture was transferred to the ESR spectrometry cell, and the DMPO–OH spin adduct (derived from ·OH) was quantified after 60 s on the X-band ESR spectrometer using 20 μM TEMPOL as the standard (similarly to the case with DMPO–OOH quantification). The measurement conditions for ESR were as follows: field sweep, 330.5–340.5 mT; field modulation frequency, 100 kHz; field modulation width, 0.1 mT; amplitude, 20; sweep time, 2 min; time constant, 0.03 s; microwave frequency, 9.42 GHz; microwave power, 4 mW. In an experiment involving kinetic analysis using double reciprocal plots, different concentrations of DMPO were added to the reaction system.

### 2.5. Scavenging of the Stable Radical DPPH

An aliquot (80 μL) of different dilutions of GSL-MW or different concentrations of ascorbic acid (reference agent) was mixed with 16 μL of 100 mM Tris-HCl buffer (pH 7.5), 64 μL of 100% ethanol, and 40 μL of 1 mM DPPH dissolved in 100% ethanol in a well of a 96-well microplate. After the plate was kept in a light-shielded place for 20 min, absorbance was measured at 520 nm using a microplate reader (FilterMax F5, Molecular Devices, Inc., Sunnyvale, CA, USA). The DPPH scavenging rate was calculated using the following equation: ([A520 in the solvent control − A520 in specimen]/A520 in the solvent control) × 100, where A520 is absorbance at 520 nm.

### 2.6. hGFs, Culture Conditions, and Cell Viability Assay

The hGFs were purchased from ScienCell Research Laboratories (Carlsbad, CA, USA). Dulbecco’s modified Eagle’s medium (DMEM; Thermo Fisher Scientific, Waltham, MA, USA) supplemented with 10% fetal bovine serum (FBS; Thermo Fisher Scientific), 100 U/mL penicillin (Wako Pure Chemical Industries), and 0.1 mg/mL streptomycin (Wako Pure Chemical Industries) was used for cell culture. The cell suspension (100 μL; 2 × 10^4^ cells/mL) was placed in each well of a 48- or 96-well culture plate and incubated at 37 °C in humidified 5% CO_2_ for the designated time. The cell proliferation or cell viability was determined using the methyl thiazolyl tetrazolium (MTT) assay [13,14], where 100 or 200 μL of the MTT reagent (1 mg/mL) was added to each well of the 96- or 48-well culture plate, respectively, and subsequently, the mixture was incubated for 3 h. After removal of the MTT reagent, 150 or 250 μL of DMSO was placed in each well of the 96- or 48-well culture plate for 30 min and absorbance was measured at 560 nm using a microplate reader (Promega GloMax Discover GM 3000; Promega, Fitchburg, WI, USA).

### 2.7. Cell Proliferation of Subconfluent hGFs After 3-min Pretreatment with GSL-MW

After the cells reached subconfluence (approximately 30% confluence), they were washed with phosphate-buffered saline (PBS) and exposed to different dilutions of GSL-MW or 0.9% NaCl. After 3 min, the cells were washed with PBS and cultured in fresh medium at 37 °C in humidified 5% CO_2_ for 24 h; subsequently, the cell viability rate was determined using the MTT assay.

### 2.8. Cell Proliferation of Subconfluent hGFs Exposed to Pure Water or 100 μM Hydrogen Peroxide (H_2_O_2_)

After the cells reached subconfluent conditions (approximately 30% confluence), they were washed with PBS and then exposed to 10× and 100× dilutions of GSL-MW for 3 min. Subsequently, GSL-MW was removed, and the cells were washed with PBS and exposed to pure water or 100 μM H_2_O_2_ for 3 min. Next, after washing with PBS, the cells were incubated with fresh medium at 37 °C in humidified 5% CO_2_ for 24 h, following which the cell viability rate was determined using the MTT assay. For this assay, DMEM was used as the control for GSL-MW.

### 2.9. Cell Viability of Confluent hGFs Exposed to 12.5–100 mM Hydrogen Peroxide (H_2_O_2_)

After the cells reached confluence, they were washed with PBS and exposed to 10× and 100× dilutions of GSL-MW for 3 min. GSL-MW was removed, and the cells were washed with PBS and then exposed to 12.5, 25, 50, or 100 mM H_2_O_2_ for 10 min. Subsequently, after washing with PBS, the cells were incubated with fresh medium at 37 °C in humidified 5% CO_2_ for 24 h to determine the cell viability rate using the MTT assay. For this assay, PBS was used as the control for GSL-MW.

### 2.10. Statistical Analyses

Statistical analyses of the quantitative data for multiple groups were performed using the Tukey–Kramer multiple comparison test (JMP Pro 16 software; SAS Institute, Cary, NC, USA).

## 3. Results

### 3.1. ESR-Spin Trapping Technique for Determination of Superoxide Anion Radicals (O_2_^•−^) Generated by the HPX–XOD Cell-Free System

**Figure 1:**
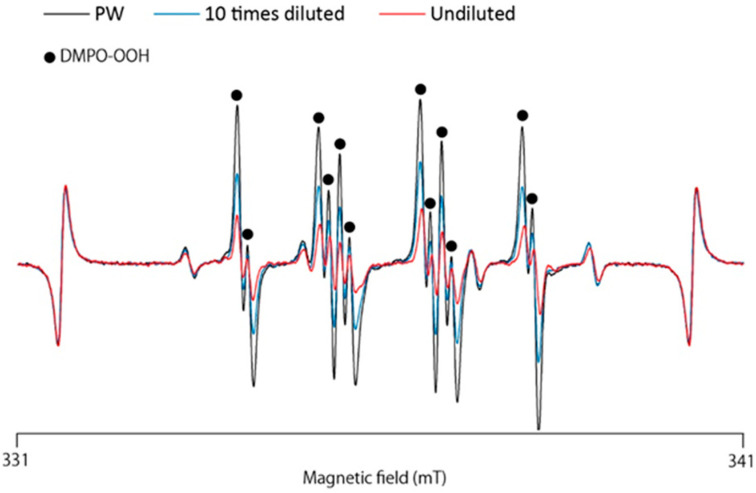
Representative ESR spectra of DMPO–OOH obtained from the hypoxanthine (HPX)–xanthine oxidase (XOD) reaction with pure water (PW), 10× diluted, and undiluted GSL-MW.

**Figure 2:**
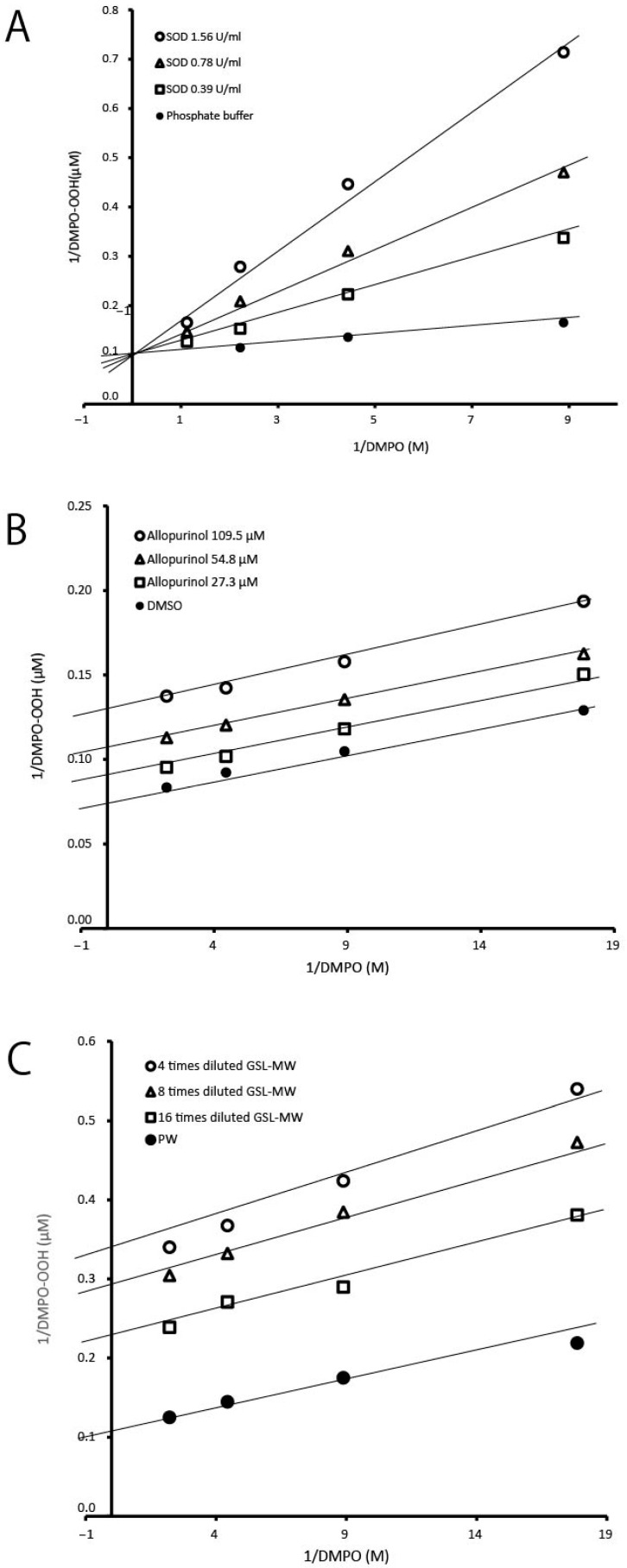
Double-reciprocal plots showing formation of DMPO–OOH versus different concentrations of DMPO at different concentrations of superoxide dismutase (SOD) (**A**), allopurinol (**B**), and GSL-MW (**C**).

Using the ESR-spin trapping technique, we examined whether GSL-MW could scavenge O_2_^•−^ generated via the HPX–XOD reaction. When a spin trap, DMPO, was added to the HPX–XOD reaction system, an ESR signal with hyperfine coupling constants of aN = 1.37 mT, aHβ = 1.10 mT, and aHγ = 0.12 mT, which were assigned to DMPO–OOH (spin adduct of DMPO and O_2_^•−^) [15], was observed. Figure 1 shows the representative ESR spectra of the pure water control and different dilutions of GSL-MW, showing that the signal intensity of DMPO–OOH reduced by treatment with GSL-MW in a dilution-dependent manner. In this system, the reduction of the DMPO–OOH signal intensity is caused by O_2_^•−^ scavenging and/or interference with the HPX–XOD reaction [12,16]. Thus, to confirm whether GSL-MW interfered with the enzymatic reaction of HPX–XOD, the competitive reaction between DMPO and GSL-MW or the reference agents was examined by constructing double-reciprocal plots (corresponding to Lineweaver–Burk plots, used to represent enzyme kinetics). Figure 2 shows the double-reciprocal plots for SOD as an authentic O_2_^•−^ scavenger, allopurinol as a XOD inhibitor [17], and GSL-MW. In the SOD plots, a linear pattern, with an intersection on the y axis denoting competitive scavenging for O_2_^•−^ with DMPO, was obtained. In the allopurinol plots, a parallel linear pattern denoting interference with the HPX–XOD reaction was obtained. In the GSL-MW plots, similar to that observed for allopurinol, a parallel linear pattern was observed, suggesting that the inhibition of DMPO–OOH formation by GSL-MW in the HPX–XOD system was attributable to an interaction of GSL-MW with the enzymatic reaction.

### 3.2. ESR-Spin Trapping Technique for Determination of Superoxide Anion Radicals (O_2_^•−^) Generated by the Photoexcitation of Riboflavin

**Figure 3:**
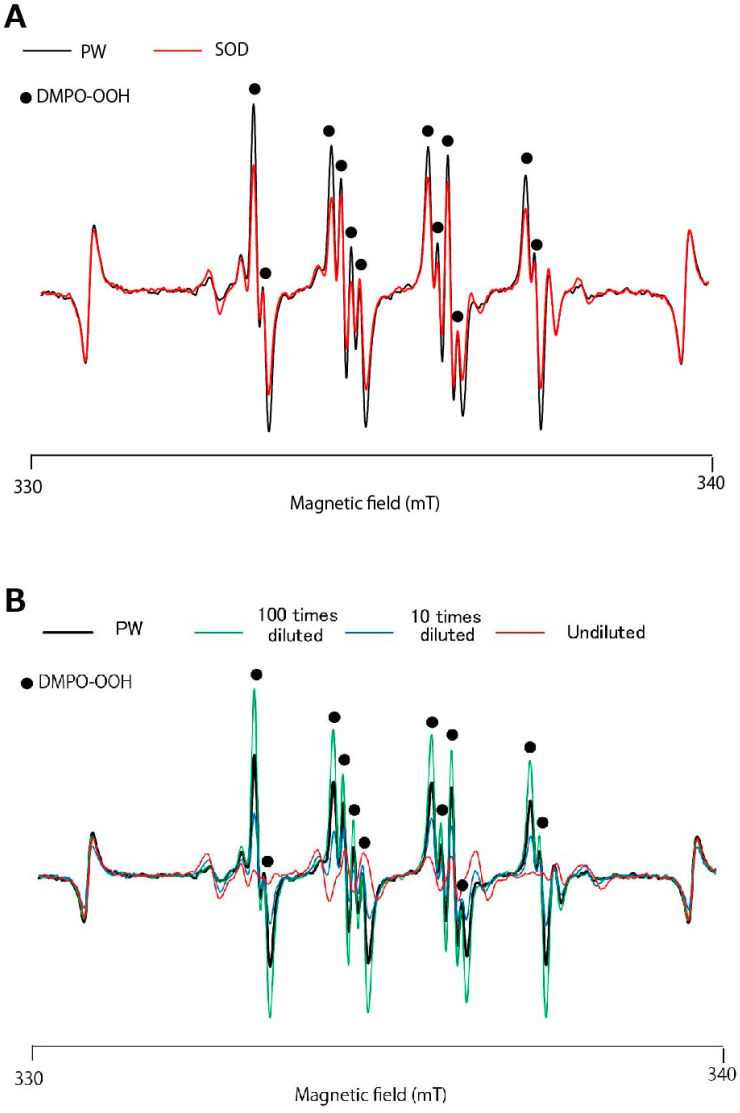
Representative ESR spectra of DMPO–OOH obtained from the photoexcitation of riboflavin. (**A**) Spectra obtained from the samples treated with pure water (PW) and superoxide dismutase (SOD; final concentration, 0.59 U/mL); (**B**) Spectra obtained from the samples with treated with PW and 100× diluted, 10× diluted, and undiluted GSL-MW.

As shown in Figure 2, the decrease in O_2_^•−^ caused by GSL-MW in the HPX–XOD system was attributable to an interaction with the enzymatic reaction. Therefore, we used the photoexcitation of riboflavin as another O_2_^•−^ generation system. Figure 3A shows the representative ESR spectra of the pure water control and SOD (final concentration, 0.59 U/mL), revealing that the signal intensity of DMPO–OOH, estimated based on the height of the first peak, was reduced by approximately one-fourth by SOD compared to that of pure water. In the case of GSL-MW (Figure 3B), undiluted and 10× diluted GSL-MW caused 92 and 24% reduction in DMPO–OOH signal intensity, respectively. In contrast, 100× dilution of GSL-MW resulted in a 67% increase in DMPO–OOH signal intensity. In this assay, the signals of DMPO-OOH spectra around higher magnetic field became weaker than those around the lower magnetic field possibly due to much shorter half-life of DMPO-OOH compared to that of DMPO-OH [18,19]. As such, the first signal intensity at the lower magnetic field was estimated for DMPO-OOH yield. The assay was performed three times per each sample, and almost identical spectra were obtained in each sample.

### 3.3. ESR-Spin Trapping Technique for Determinations of Hydroxyl Radicals (·OH) Generated by Fenton Reaction

**Figure 4:**
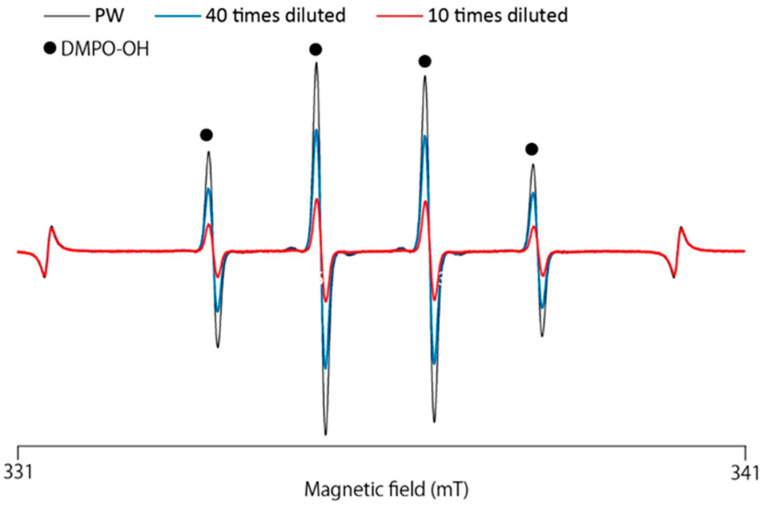
Representative ESR spectra of DMPO–OH obtained from the Fenton reaction in the presence of pure water (PW) and 40× and 10× diluted GS.

**Figure 5:**
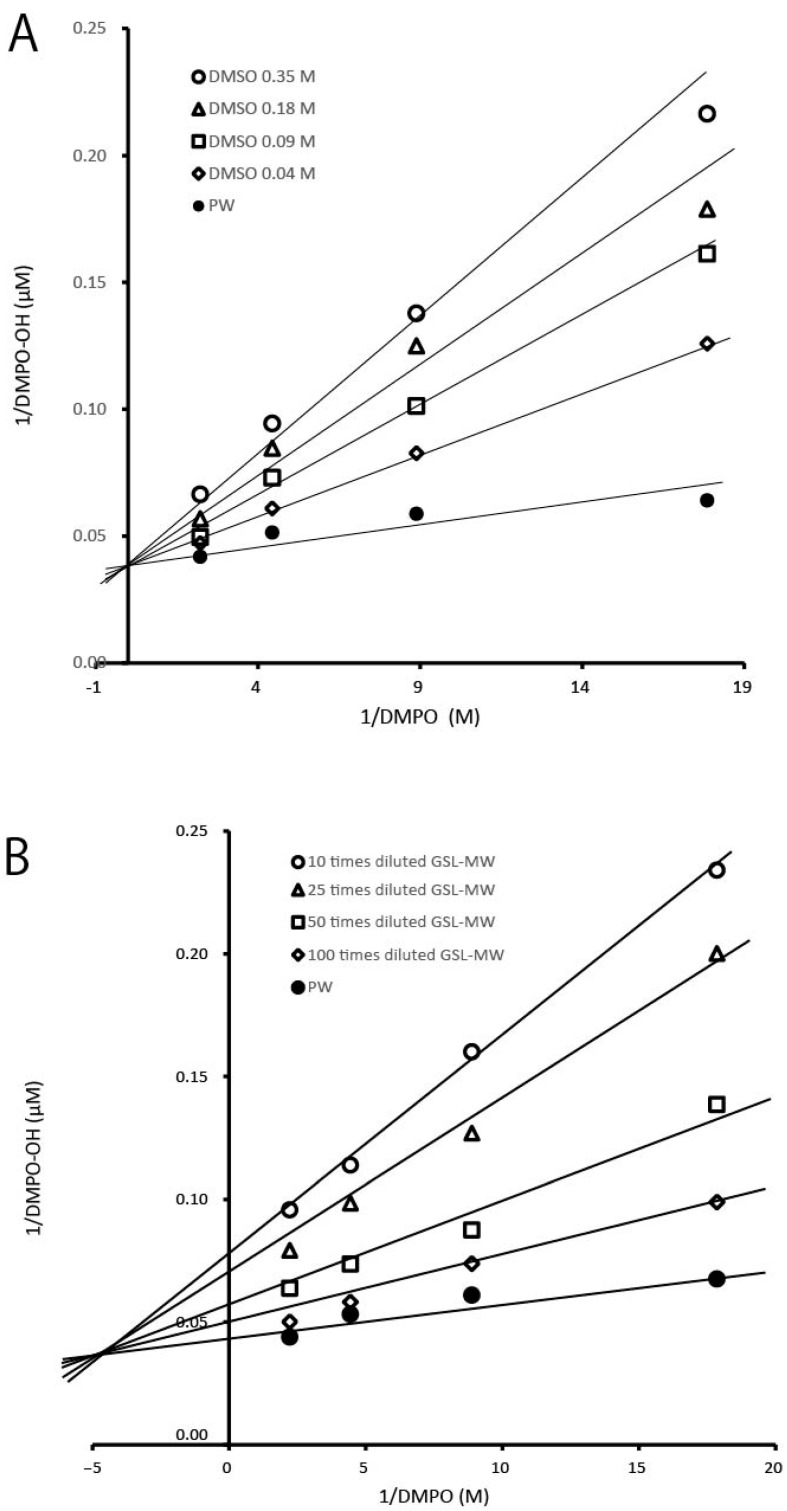
Double-reciprocal plots showing formation of DMPO–OH versus different concentrations of DMPO at different concentrations of dimethyl sulfoxide DMSO (**A**) and GSL-MW (**B**).

We used the ESR-spin trapping method to determine whether GSL-MW has the ability to scavenge ·OH generated by the Fenton reaction. When DMPO was added to the reaction mixture, an ESR signal with hyperfine coupling constants of aN = 1.49 and aH = 1.49 mT, which were assigned to DMPO–OH (a spin adduct of DMPO and OH) [15], was observed. Figure 4 shows the representative ESR spectra of the pure water control and different dilutions of GSL-MW, showing that the signal intensity of DMPO–OH was reduced by GSL-MW in a dilution-dependent manner. Similar to that observed in the HPX–XOD system, the reduction of the DMPO–OH signal intensity could be caused by ·OH scavenging and/or interference with the Fenton reaction. To confirm whether GSL-MW interferes with the Fenton reaction, the competitive reaction between DMPO and GSL-MW or a reference agent, DMSO (a ·OH scavenger) [20,21,22], was examined by constructing double-reciprocal plots. Figure 5 shows double-reciprocal plots for DMSO and GSL-MW. In the DMSO plots, a linear pattern whose intersection was on the y axis, denoting competitive scavenging of ·OH by DMPO, was obtained. In the GSL-MW plots, the linear pattern intersection was shifted to the negative side of the x axis, suggesting that DMPO–OH formation was inhibited not only due to ·OH scavenging but also due to interference with the Fenton reaction. Regarding the dilution-dependent effect of GSL-MW on the reduction of DMPO–OH spectra, pH values seemed unlikely to have affected the results, since pH values of 10× and 100× GSL-MW measured by a glass electrode method were identical to 8.6. To ascertain if transition metals such as iron (Fe) and copper (Cu) existed at enough levels to induce a Fenton reaction, we checked the reaction of 1×–1000× diluted GSL-MW and H_2_O_2_ by the ESR-spin trapping method and found no DMPO–OH spectra, indicating that the effects of transition metals could be ignored.

### 3.4. Scavenging of the Stable Radical DPPH

**Figure 6:**
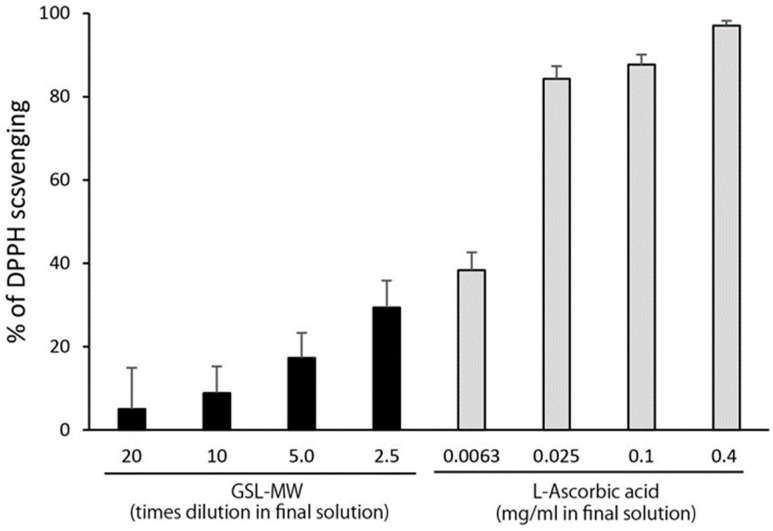
Scavenging of 2,2-diphenyl-1-picrylhydrazyl (DPPH) by GSL-MW and L-ascorbic acid. Each value represents the mean + standard deviation (n = 6).

The scavenging of DPPH by GSL-MW and L-ascorbic acid is shown in Figure 6. GSL-MW and L-ascorbic acid scavenged DPPH in a dilution/concentration-dependent manner. The effect of GSL-MW was, however, less potent than that of L-ascorbic acid because the effect of 0.1 mg/mL of L-ascorbic acid was approximately three times more potent than that of 2.5× diluted GSL-MW (the lowest dilution rate).

### 3.5. Cell Proliferation After 3-min Pretreatment with GSL-MW

**Figure 7:**
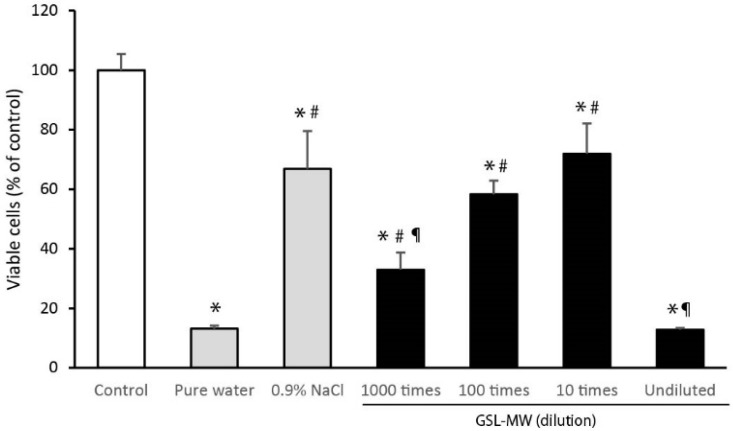
Proliferative response of subconfluent human gingival fibroblasts (hGFs) after 3-min pretreatment with different dilutions of GSL-MW. Cell viability was determined 24 h after the pretreatment. Each value represents the mean + standard deviation (n = 7). *, #, and ¶ represent significant difference (*p* < 0.05) from the data obtained from control, pure water, and 0.9% NaCl samples, respectively.

Figure 7 shows the proliferative response of subconfluent hGFs 24 h after pretreatment with diluted GSL-MW, pure water, or 0.9% NaCl for 3 min. Pretreatment with pure water resulted in a low level of viable cells owing to osmolality-related cellular damage. Pretreatment with 0.9% NaCl reduced the level of viable cells by 33% compared to that observed in the control without pretreatment. In the case of GSL-MW, the levels of viable cells 24 h after pretreatment with 10× and 100× dilutions of GSL-MW were similar to that observed after pretreatment with 0.9% NaCl. Pretreatment of cells with undiluted GSL-MW reduced viable cells to a level as low as that observed after pretreatment with pure water; the level of viable cells after pretreatment with 1000× diluted GSL-MW was significantly higher than that observed after pretreatment with pure water, but lower than that observed after pretreatment with 0.9% NaCl.

### 3.6. Cell Proliferation After Exposure to Pure Water or 100 μM Hydrogen Peroxide (H_2_O_2_)

**Figure 8:**
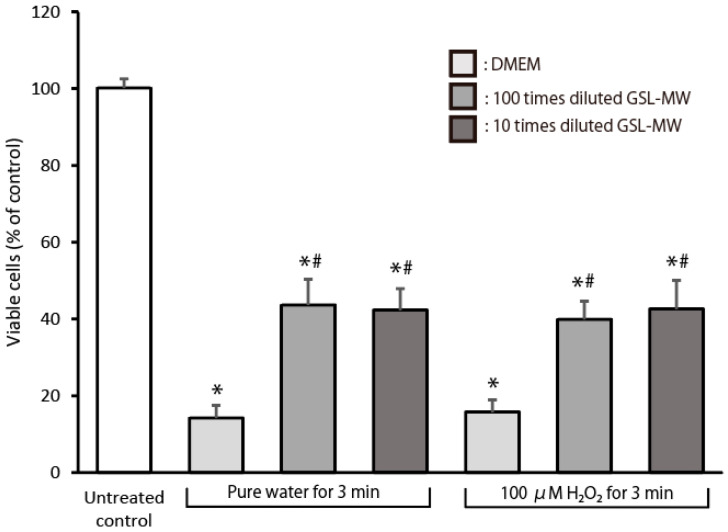
Proliferative response of subconfluent hGFs, pretreated for 3 min with 10× and 100× dilutions of GSL-MW, after 3-min exposure to pure water or 100 μM hydrogen peroxide (H_2_O_2_). Cell viability was determined 24 h after exposure to pure water and 100 μM H_2_O_2_. Each value represents the mean + standard deviation (n = 6). * and # represent significant differences (*p* < 0.05) from the data obtained from untreated control and the corresponding Dulbecco’s modified Eagle’s medium (DMEM) samples, respectively.

Figure 8 shows the proliferative response of subconfluent hGFs, which were pretreated with different dilutions of GSL-MW for 3 min, 24 h after exposure to pure water or 100 μM H_2_O_2_ for 3 min. The level of viable cells 24 h after the exposure was significantly higher in the GSL-MW-pretreated cells than in the DMEM-pretreated control cells. That is, the decrease in viable cells, induced by pure water or 100 μM H_2_O_2_ exposure, was alleviated by pretreatment with 10× and 100× diluted GSL-MW.

### 3.7. Cell Viability of Confluent hGFs Exposed to 12.5–100 mM Hydrogen Peroxide (H_2_O_2_)

**Figure 9:**
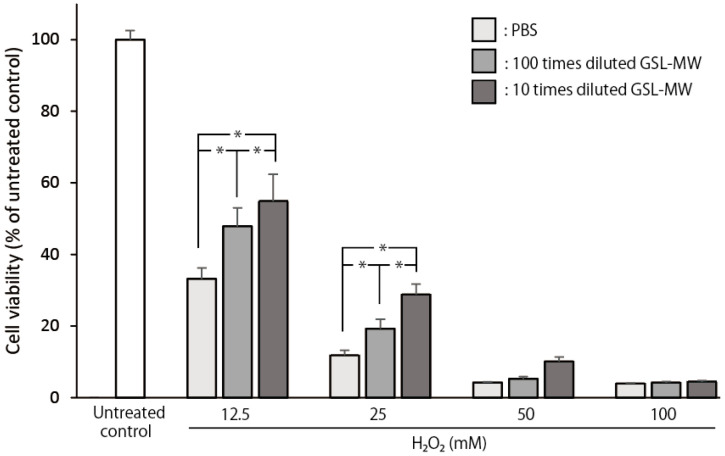
Cell viability of confluent hGFs, pretreated for 3 min with 10× and 100× dilutions of GSL-MW, after 10-min exposure to different concentrations of hydrogen peroxide (H_2_O_2_). Cell viability was determined 24 h after the H_2_O_2_ exposure. Each value represents the mean + standard deviation (n = 6). * represent significant differences (*p* < 0.05) between results for the two groups.

Figure 9 shows the cell viability of confluent hGFs, which were pretreated with different dilutions of GSL-MW for 3 min, 24 h after exposure to different concentrations of H_2_O_2_ for 10 min. When PBS-pretreated confluent cells were exposed to 12.5, 25, 50, and 100 mM H_2_O_2_, the cell viability reduced to 67, 88, 96, and 96%, respectively, compared to that in the untreated control group. Pretreatment with 10× and 100× diluted GSL-MW significantly hindered the decrease in cell viability caused by exposure to 12.5 and 15 mM H_2_O_2_ but could not alleviate the decrease in cell viability caused by exposure to 50 and 100 mM H_2_O_2_.

## 4. Discussion

In this study, we examined the radical scavenging activity of processed mineral water derived from the Great Salt Lake (GSL-MW). Analysis using the ESR-spin trapping technique for detection of O_2_^•−^ generated by the HPD–XOD reaction revealed that diluted GSL-MW reduced the signal intensity of DMPO–OOH (the spin adduct of O_2_^•−^ and DMPO), suggesting that GSL-MW could scavenge O_2_^•−^. To confirm this hypothesis, a kinetic study was conducted using double-reciprocal plots, which revealed that GSL-MW interfered with the HPD–XOD reaction, in a manner similar to that shown by the XOD inhibitor allopurinol. Thus, we used the photoexcitation of riboflavin as another O_2_^•−^ generation system. The resultant DMPO–OOH signal intensity was reduced by undiluted and 10× diluted GSL-MW but was increased by 100× diluted GSL-MW. One of the reasons for this inconsistency in the findings seems to be the interactions between minerals in GSL-MW (Table 1) and EDTA·3Na used in the reaction system; this is because EDTA acts as a chelating agent that forms a stable water-soluble chelate with metal ions having valency greater than two. Since the width of ESR spectra of GSL-MW added samples were a bit varied between the dilutions (Figure 3B) and dissolved oxygen is known to broaden ESR spectrum [23,24], we checked dissolved oxygen concentrations in the GSL-MW dilutions but clear relation was not found between the width of ESR spectra and the dissolved oxygen concentrations (4.5, 4.8, and 5.1 mg/L for 1×, 10×, and 100× diluted GSL-MW, respectively, as measured by a diaphragm polarographic method). In the present study, we could not find conclusive evidence to prove that GSL-MW could scavenge O_2_^•−^. Regarding the ·OH scavenging capacity determined by the Fenton reaction and the ESR-spin trapping technique, the signal intensity of DMPO–OH (the spin adduct of ·OH and DMPO) was reduced by 10× and 40× diluted GSL-MW. To ascertain if GSL-MW has the ability to scavenge ·OH, a kinetic analysis was conducted using double-reciprocal plots, and the results revealed that GSL-MW not only scavenged ·OH directly but also interfered with the Fenton reaction that generated ·OH. In addition to the ·OH scavenging capacity, GSL-MW scavenged DPPH, a stable radical, although the scavenging ability of GSL-MW seemed much weaker than that of L-ascorbic acid. To summarize the results obtained so far, GSL-MW scavenged ·OH and, albeit weakly, DPPH, but whether GSL-MW could scavenge O_2_^•−^ remains uncertain. The mechanism by which GSL-MW scavenges ·OH and, albeit weakly, DPPH, is not clear at present. Although several studies have reported the antioxidant properties of mineral water [1,2,3,4,5,6], few studies have been conducted to examine the radical scavenging activity of mineral water. Copper (Cu), magnesium (Mg), selenium (Se), and zinc (Zn) are antioxidant minerals [25,26,27,28], and Mg is a major component of GSL-MW (Table 1). Redox-inactive metal ions such as Mg^2+^ have been shown to markedly accelerate the radical-scavenging reactions of antioxidants [29,30,31], despite the fact that few studies have been conducted to analyze the radical-scavenging activity of Mg. Mg may be associated with the radical scavenging capacity of GSL-MW. Further studies are required to elucidate the fundamental antioxidant characteristics of GSL-MW.

Our previous studies showed the cytoprotective effects of short-term treatment (1–3 min) with a potent antioxidant on hGFs [7,8], with the aim of improving oral health. These studies have shown that antioxidants can scavenge free radicals and protect hGFs exposed to harsh environmental conditions, such as pure water, H_2_O_2_, and serum-free medium. In this study, we examined whether short-term treatment with GSL-MW could protect hGFs. As shown in Figure 6, the cytotoxic effects of 10× and 100× dilutions of GSL-MW on subconfluent hGFs were similar to those of 0.9% NaCl; therefore, these two dilutions of GSL-MW were used in the subsequent experiments, which revealed the cytoprotective action of the two GSL-MW dilutions, as shown in Figure 7 and Figure 8. As shown in Table 2, the osmolality of 100× diluted GSL-MW was lower than that of 0.9% NaCl and that of 10× diluted GSL-MW was higher than that of 0.9% NaCl. Thus, the proposed cytoprotective effects of 10× and 100× dilutions of GSL-MW were unlikely to be related to osmolality. As shown in Figure 7, 3-min pretreatment with 10× and 100× dilutions of GSL-MW alleviated the decrease in the level of viable hGFs that were exposed to pure water or 100 μM H_2_O_2_. Cellular damage caused by pure water was most likely due to hypo-osmotic stress, indicating that GSL-MW may have the potential to enhance cell membrane stability. Regarding cellular damage caused by 100 μM H_2_O_2_, the cell viability of hGFs exposed to 100 μM H_2_O_2_ decreased to a level similar to that observed after exposure of hGFs to pure water, suggesting that cellular damage after exposure to 100 μM H_2_O_2_ was caused by hypo-osmotic stress, similar to that observed after exposure to pure water. Regarding the possible involvement of ROS in hypoosmotic stress, stress conditions have been reported to accelerate ROS formation, possibly through intracellular Ca^2+^ sparks in mammalian muscle fibers and cardiomyocytes [32,33,34]; this suggests that the cytoprotective effects of the two GSL-MW dilutions might be attributable to their antioxidative action. Thus, further experiments were conducted using concentrations of H_2_O_2_ higher than 100 μM to examine the cytoprotective effects of GSL-MW, as shown in Figure 8. Pretreatment with 10× and 100× dilutions of GSL-MW alleviated the cellular damage caused by H_2_O_2_-induced oxidative stress, suggesting that the cytoprotective effects of GSL-MW could be attributed to its antioxidative potential.

This study had some limitations. First, the components of GSL-MW responsible for its radical scavenging activity are currently unclear. Second, the direct relationship between the radical scavenging activity of GSL-MW and its cytoprotective effects remains unclear. Third, since the in vivo efficacy of GSL-MW has not been established in animal models and/or humans, we lack definitive evidence to prove that GSL-MW can be used for improvement of oral health.

Despite the limitations of this study, our findings show that GSL-MW has antioxidant potential and cytoprotective effects on hGFs, suggesting that GSL-MW can be used for maintaining oral health. We will further examine how GSL-MW affects the oral environment by overcoming these limitations.

## 5. Conclusions

This study revealed that GSL-MW has the ability to scavenge free radicals in some extent, and the pretreatment of GSL-MW is able to protect hGFs from harsh environmental conditions such as exposure to hypoosmotic stress induced by pure water and oxidative stress by H_2_O_2_. These results suggest that GSL-MW could be a useful agent to maintain a healthy oral environment.

## Figures and Tables

**Table 1:** Mineral composition of Great Salt Lake-derived processed mineral water (GSL-MW).

Mineral	Concentration (mg/mL)
Chloride (Cl)	275
Magnesium (Mg)	101
Sulfate (SO_4_)	21.4
Sodium (Na)	3.1
Potassium (K)	2.1

**Table 2:** Osmolality of GSL-MW dilutions.

	Osmolality (mOSM/kg)
Physiological saline (0.9% NaCl)	289
Undiluted GSL-MW	12,347
10-times diluted GSL-MW	1282
100-times diluted GSL-MW	118
1000-times diluted GSL-MW	13

## Data Availability

The data presented in this study are available upon request from the corresponding author.
